# Reconsidering lactate as a sepsis risk biomarker

**DOI:** 10.1371/journal.pone.0185320

**Published:** 2017-10-03

**Authors:** John L. Moran, John Santamaria

**Affiliations:** 1 Department of Intensive Care Medicine, The Queen Elizabeth Hospital, Woodville, South Australia, Australia; 2 Department of Critical Care Medicine, St Vincent’s Hospital Melbourne, Fitzroy, Victoria, Australia; Institut d'Investigacions Biomediques de Barcelona, SPAIN

## Abstract

**Objectives:**

There has been renewed interest in lactate as a risk biomarker in sepsis and septic shock. However, the ability of the odds ratio (OR) and change in the area under the receiver operator characteristic curve (AUC-ROC) to assess biomarker added-value has been questioned.

**Design, setting and participants:**

A sepsis cohort was identified from the ICU database of an Australian tertiary referral hospital using APACHE III diagnostic codes. Demographic information, APACHE III scores, 24-hour post-admission patient lactate levels, and hospital mortality were accessed.

**Measurements and main results:**

Hospital mortality was modelled using a base predictive logistic regression model and sequential addition of admission lactate, lactate clearance ([lactate^admission^—lactate^final^]/lactate^admission^), and area under the lactate-time curve (LTC). Added-value was assessed using lactate index OR; AUC-ROC difference (base-model versus lactate index addition); net (mortality) reclassification index (NRI; range -2 to +2); and net benefit (NB), the number of true positives per patient adjusted for the number of false positives. The data set comprised 717 patients with mean(SD) age and APACHE III score 61.1(16.5) years and 68.3(28.2) respectively; 59.2% were male. Admission lactate was 2.3(2.5) mmol/l; with lactate of ≥ 4 mmol/L (37% hospital mortality) in 17% and patients with lactate < 4 mmol/L having 18% hospital mortality. The admission base-model had an AUC-ROC = 0.81 with admission lactate OR = 1.127 (95%CI: 1.038, 1.224), AUC-ROC difference of 0.0032 (-0.0037, 0.01615; P = 0.61), and NRI 0.240(0.030, 0.464). The over-time model had an AUC-ROC = 0.86 with (i) clearance OR = 0.771, 95%CI: 0.578, 1.030; P = 0.08; AUC-ROC difference 0.001 (-0.003, 0.014; P = 0.78), and NRI 0.109(-0.193, 0.425) and (ii) LTC OR = 0.997, 95%CI: 0.989, 1.005, P = 0.49; AUC-ROC difference 0.004 (-0.002, 0.004; P = 0.34), and NRI 0.111(-0.222, 0.403). NB was not incremented by any lactate index.

**Conclusions:**

Lactate added-value assessment is dependent upon the performance of the underlying predictive model and should incorporate risk reclassification and net benefit measures.

## Introduction

The recent interest in the role of lactate as a biomarker of risk in the critically ill and in sepsis and septic shock in particular [1] is perhaps surprising, given the long history of such observations [2], a point reiterated in commentaries [3, 4]. The landmark trial of early goal-directed therapy (EGDT [5]) by Rivers, Nguyen and co-workers (2001) and the failure of three large multi-centre trials (2014–2015) [6–8] to confirm these findings have possibly refocused the attention of investigators on hyperlactataemia.

The statistical methods used in the assessment of lactate as a biomarker in sepsis [9] have been calculation of the effect size (as odds ratio (OR)) and statistical significance of lactate as single or multiple lactate measurements over the first 24 hours, or clearance over a specified time frame (commonly 2 or 6 hours), in either univariate or multivariate logistic models [10–13]; and the difference in the area under the receiver operator characteristic curve (AUC-ROC) of competing models. However, biomarker assessment or its “added value” has recently been intensely debated. The ability of the OR to “meaningfully describe a marker’s ability to classify subjects” has been questioned [14] and “testing ROC areas generated from nested models”, that is models with and without the biomarker, is “an approach with serious validity problems” [15] and amounts to “…literally testing the same null hypothesis twice” [16]. Authors have also not explained the exact clinical import of increments of the area under the receiver operator characteristic curve (AUC-ROC) at, say, the second decimal place; that is, is this small improvement “worthwhile”? [17, 18].

With the above caveats in mind, we undertook analysis of the added value of lactate as a risk [19, 20] biomarker, with respect to in-hospital mortality, in patients with sepsis and septic shock using prospectively recorded data from a tertiary level general Australian intensive care unit (ICU). We report conventional indices of biomarker assessment, OR and AUC-ROC; and measures recently recommended in the TRIPOD statement [21]: indices of risk re-classification, the integrated discrimination improvement index (IDI) and the net reclassification index (NRI) [20, 22]; and measures of net benefit, derived from decision curve analysis [20, 23]. Given that the data are from a single ICU, the thrust of the paper is methodological. However, we do not eschew clinical comment and reflections on lactate as a guide to therapy (lactate as a predictive biomarker [24]), although the latter is not to be confused with determination of lactate as a prognostic risk biomarker [25].

## Methods

### Data acquisition

St Vincent's Hospital Melbourne in Victoria is a 400-bed university affiliated tertiary referral hospital. The single intensive care unit of 20 beds admits approximately 1700 patients each year and they include those undergoing cardiac surgery and neurosurgery. Patient observations are prospectively entered within a clinical information system (IntelliSpace Critical Care and Anesthesia, Philips) which also imports the results of routine biochemical and haematology tests. In addition, detailed patient information is entered within a second database that provides information to the Australian and New Zealand Intensive Care Society (ANZICS) adult patient database [26], the latter using an Australian modification of the APACHE III diagnostic codes [27]. This patient database has demographic information, severity of illness scores (APACHE III [28]), Charlson Comorbidity score [29] and outcomes of ICU and hospital discharges. Both data sources were used to extract patient details and relevant pathology results for those patients coded with sepsis or septic shock (diagnosis codes 501–504) as the primary diagnosis. This study was approved as a quality assurance activity by the St Vincent's Hospital Melbourne Quality and Risk Department. All data was anonymized and de-identified before researcher access and neither author was involved in data anonymization.

### Statistical analysis

Continuous variables were reported as mean(SD) and statistical significance was ascribed at P ≤ 0.05; analysis was conducted using Stata^™^ V14.2 (2016, College Station, TX) and R statistical software (V 3.3.1).

The overall modelling process is shown in Table 1:

**Table 1:** 

Model	Functionals	Development
Initial logistic	Non-linear covariate effects (fractional polnomials)	Information criteria: AIC, BIC
	Interactions	Discrimination: AUC-ROC
	Collinearity check	Calibration: Hosmer-Lemeshow test
		Polynomial calibration plots
		In-sample and out-of-sample predictive bias
		Overfitting
Lactate added		
Lactate form	Initial lactate	
	Fractional lactate clearance: (admission-final)/admission	
	Area under lactate-time curve	
	Sensitivity analysis:	
	Lactate change: admission-final	
	Lactate ratio: final/admission	
	Log lactate ratio: log(final/admission)	
Added value		
	AUC-ROC difference	
	Net reclassification index (NRI)	Bootstrapped confidence intervals
	Integrated discrimination improvement index (IDI)	Bootstrapped confidence intervals
	Net benefit

1. The modelling process was considered in two stages: a base logistic model for hospital outcome was developed with particular attention paid to the functional form of continuous variables (using fractional polynomials [30]); interactions (or effect modifiers [13]); collinearity between candidate predictors using the condition number (in non-linear models, values > 15) and the correlation between variables (rho > 0.8) [31]; and, in view of possible non-linear covariate form and the collection of data over a number of years, the potential for overfitting, or shrinkage statistics (determined by in-sample and out-of-sample predictive bias and overfitting, expressed in percentages [32, 33]). Model development was guided by progressive reduction of information criteria (Akaike (AIC) and Bayesian (BIC) information criteria [34]); the conventional criteria of discrimination (AUC-ROC) and calibration (Hosmer-Lemeshow statistic [35] and model variable parsimony.
  Calibration plots (observed binary responses versus predicted probabilities) were undertaken using 'givitiR' [36, 37], a user written package within R statistical software [38]. The relationship of predictions to the true probabilities of the event was formulated with a second logistic regression model, based upon a polynomial transformation of the predictions, the degree of the polynomial (beginning with second order) being forwardly selected on the basis of a sequence of likelihood ratio tests. The calibration belt presents 80% and 95% confidence levels; the deviation of the calibration belt from the line of identity is indicated by a reported P value.
  Categorical variables were parameterised as indicator variables including calendar years; the latter were included in all models.
2. the primary analysis followed the literature examples and addressed initial lactate (mmol/L), fractional lactate clearance ([lactate^admission^—lactate^final^]/lactate^admissionl^) [39] and area under the lactate-time curve [5], calculated as per Jaki and Wolfsegger, using the “PK” module [40] in R statistical software. We were concerned to avoid the confounding effect of dynamic [12] lactate indices (“change scores” [41]) that were related to initial lactate. We also considered: lactate change (lactate^admission^—lactate^final^), lactate ratio (lactate^final^ / lactate_admission_) and log ratio (log(lactate^final^ / lactate_admission_) = log(lactate^final^)-log(lactate^initial^)) [42]. Diagnostic measures were scatter plots of lactate clearance, change, ratio and log ratio against initial lactate; computation of Kaiser’s R (R > 1 favours change; R < 1 favours fractional lactate clearance [43]); and use of Bland-Altman plots via the user written Stata^™^ module “concord” [44] (favoured index having the minimum slope of the reduced major axis of the difference between indices versus the mean of indices).
3. The added value [20] of indices was computed using:
  - AUC-ROC difference (model with and without the marker) using bootstrap 95% intervals (n = 1000).
  - The NRI (theoretical range -2 to +2) computed by assessing the change (movement “up” or “down” within categories) in the classification of the risk / probability of patients with respect to the end point (hospital mortality) by the addition of the new marker in question; that is, NRI = *P*(up|event) − *P*(down|event) + P(down|nonevent)-P(up|event). In the absence of understandable and well-verified risk categories, a category-free (“continuous”) version may be computed, as the NRI has been demonstrated to be computationally sensitive to the number of risk categories used [45]. Furthermore, as we were interested in risk across the whole spectrum (0 to 1), we report the category-free form of NRI (NRI(>0)). The latter is a measure of the effect size of a new predictor with respect to prediction models, rather than the difference in performance of the two models [46].
    The IDI, a complement to the AUC-ROC, is defined as: IDI = (ISnew − ISold) − (IPnew − IPold), where IS is the integral of sensitivity over all possible cut-off values and IP is the corresponding integral of “1-specificity” [47]. The IDI magnitude indicates the increase in the separation of mean predicted risks/probabilities for events and non-events that has occurred by the incorporation of the new biomarker [48] and is identical to the difference in Pearson R^2^ values [20].
    Bootstrap 95% CI (n = 1000) of both NRI and IDI for event, non-event and overall are reported as opposed to P-values [49, 50]. The indices in a. and b. above were computed using the user written “incrisk” Stata^™^ module [51].
  - Net benefit, the number of true positives per patient adjusted for the number of false positives, that is:
    $$\text{Net}\;\text{Benefit}\;=\frac{\text{True}\;\text{positives}-\text{False}\;\text{positives}\;\left(\frac{p_{t}}{1-p_{t}}\right)}{n},$$ (1)
    where *n* is the total sample size and *p*_*t*_ is the probability threshold, using the written “dca” Stata^™^ module [52]. The graphical display format is of net benefit versus threshold probabilities (0 to 1), where the latter indicates potential points of risk for clinical decision making. For instance, if biomarker measurement would be undertaken at (and below) a particular patient risk(s), the X-axis may be truncated at the upper margin of plausible risk(s). As we were interested in net benefit comparisons across the whole spectrum of probabilities [53], the X-axis was maintained at 0 to 1. In the graph, the solid “Treat All” line crosses the horizontal “Treat None” line (at zero on the Y-axis) at the study prevalence value (see graphical displays below).
    Net benefit is typically used to assess the value of a diagnostic test over a range of "probability thresholds" (relative value of treatment if disease is present to value of avoiding unnecessary treatment). However, net benefit has been demonstrated to be a proper measure of model performance [54] and the highest net benefit is optimal [23, 55]. In the current paper, net benefit was used as a comparative index of model performance.

## Results

The data set (collected over 7 years) comprised 717 patients with mean(SD) age and APACHE III score 61.1(16.5) years and 68.3(28.2.2) respectively; 59.2% were male and 27% were ventilated in the first 24 hours. ICU and hospital length of stay (days) were 4.3(6.4) median 2, and 23.7(28.5) median 15, respectively. ICU and hospital mortality outcome were 12.3% and 21% respectively. The Charlson Comorbidity Index ranged from 0 to 15, median 1 and interquartile range 3. On admission lactate was 2.3(2.5) mmol/l; 17% of patients had a lactate of ≥ 4 mmol/l, with 37% hospital mortality and patients with a lactate < 4 mmol/l had a hospital mortality of 18%.

### Univariate analyses

**Fig 1:**
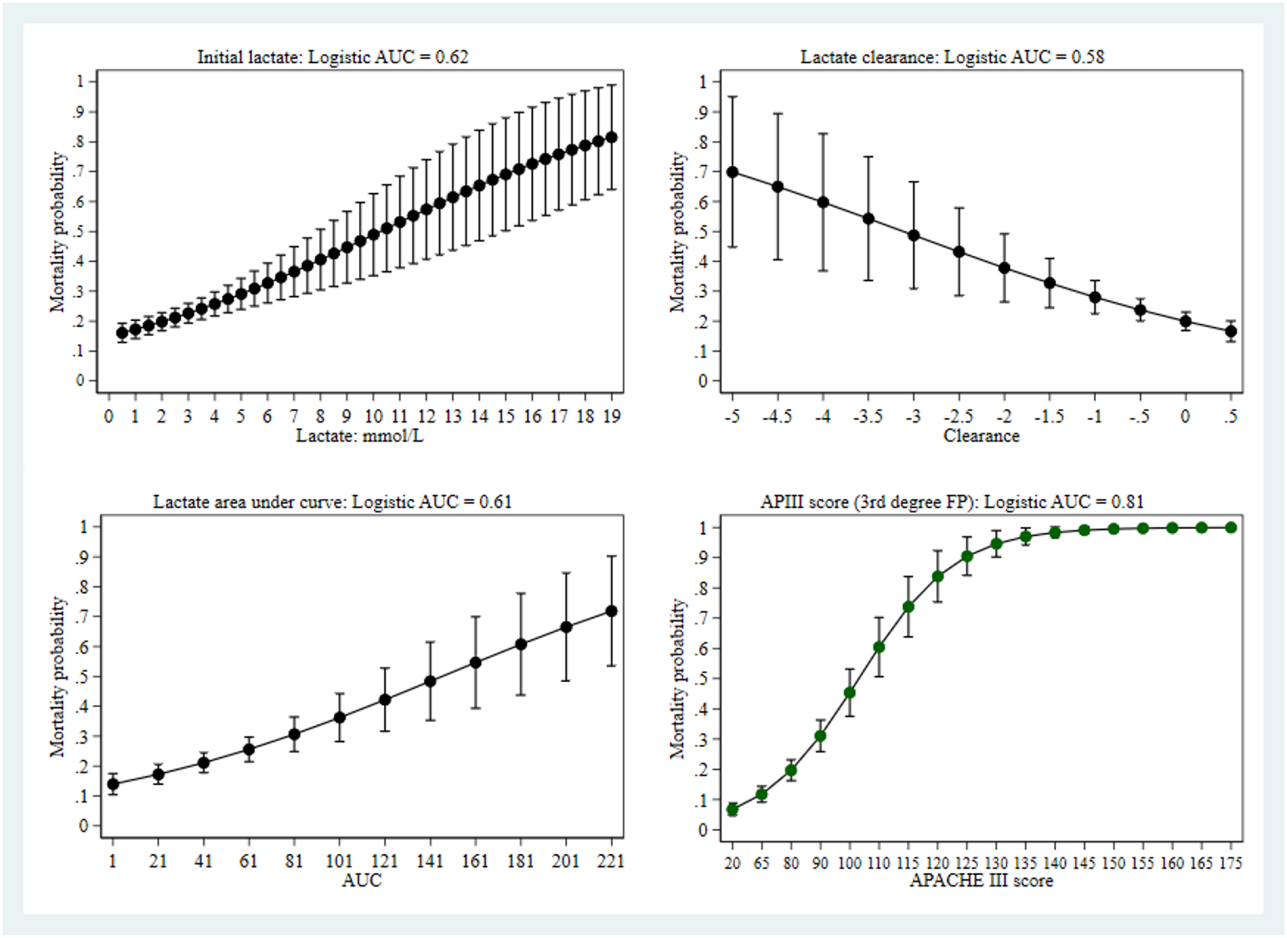
Univariate predictors of hospital mortality.

The performance of univariate predictors of hospital outcome was compared between initial lactate (OR 1.185, 95%CI: 1.049, 1.270), lactate clearance (OR 0.640. 95%CI: 0.501, 0.817), area under the lactate-time curve (OR 1.013, 95%CI: 1.008, 1.018) and APACHE III score (OR 1.053, 95%CI: 1.044, 1.063); only the latter demonstrated non-linear effect form and was parameterised as a third-degree fractional polynomial. As seen in Fig 1, all variables showed a range dependent change in mortality, with variable levels of uncertainty (95%CI). Not surprisingly, the APACHE III score, as it reflects both severity of illness and impact of therapy over 24 hours, was the best predictor with respect to the logistic AUC. Predicted probabilities from each of the logistic models showed good calibration (calibration graphs not shown), with P values ≥ 0.12.

**Fig 2:**
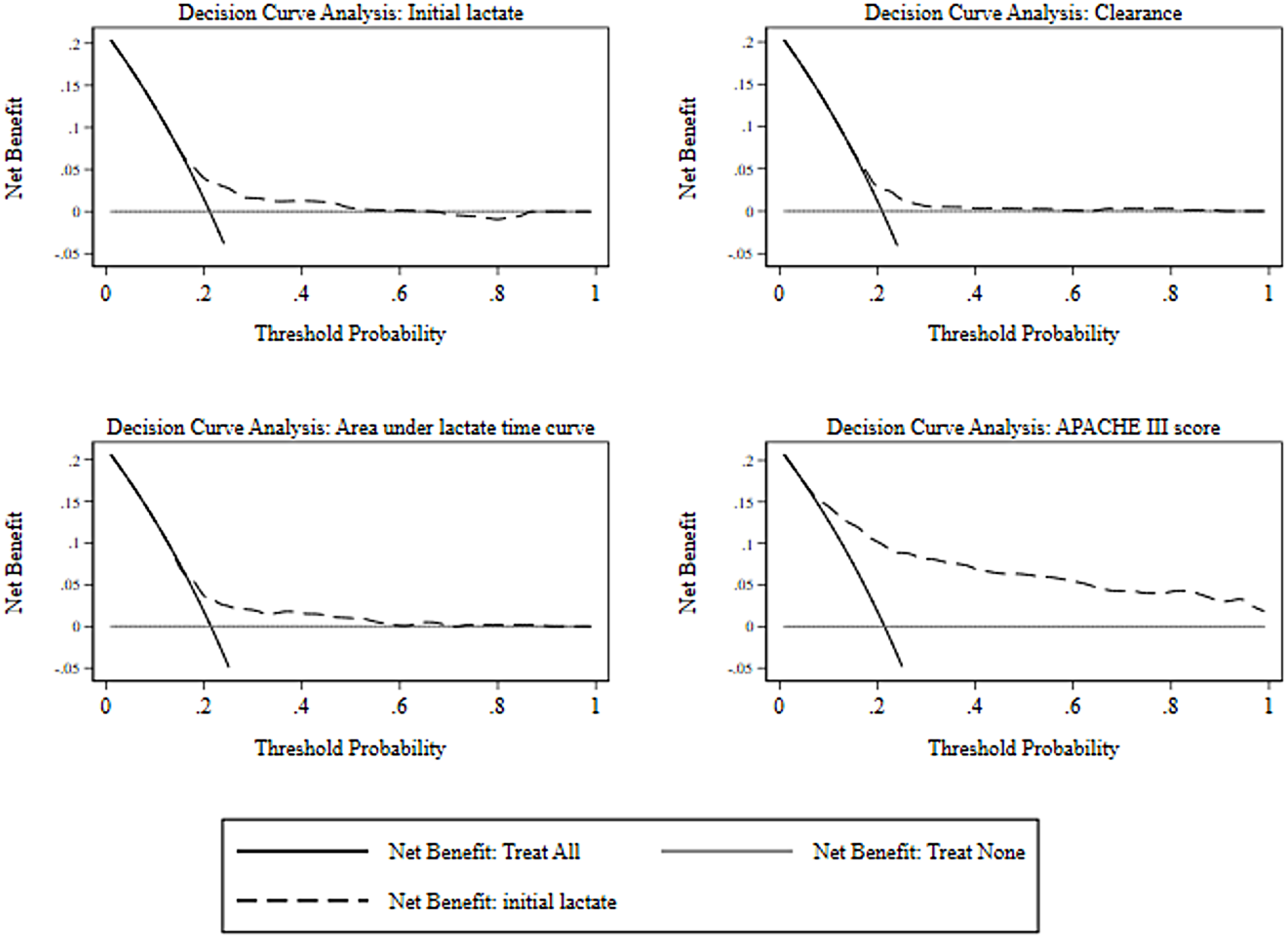
Univariate net benefit curves.

Although the logistic AUC-ROC differed between each of the predictors, a different perspective results when comparing the net benefit curves, as seen in Fig 2. There was little difference between the lactate derived indices, although net benefit of both initial lactate and area under the lactate-time-curve extends to a threshold probability of at least 0.5, compared with approximately 0.3 for clearance. Again, the net benefit of the APACHE III dominated across all threshold probabilities.

### Multivariate analysis: Admission variables

**Fig 3:**
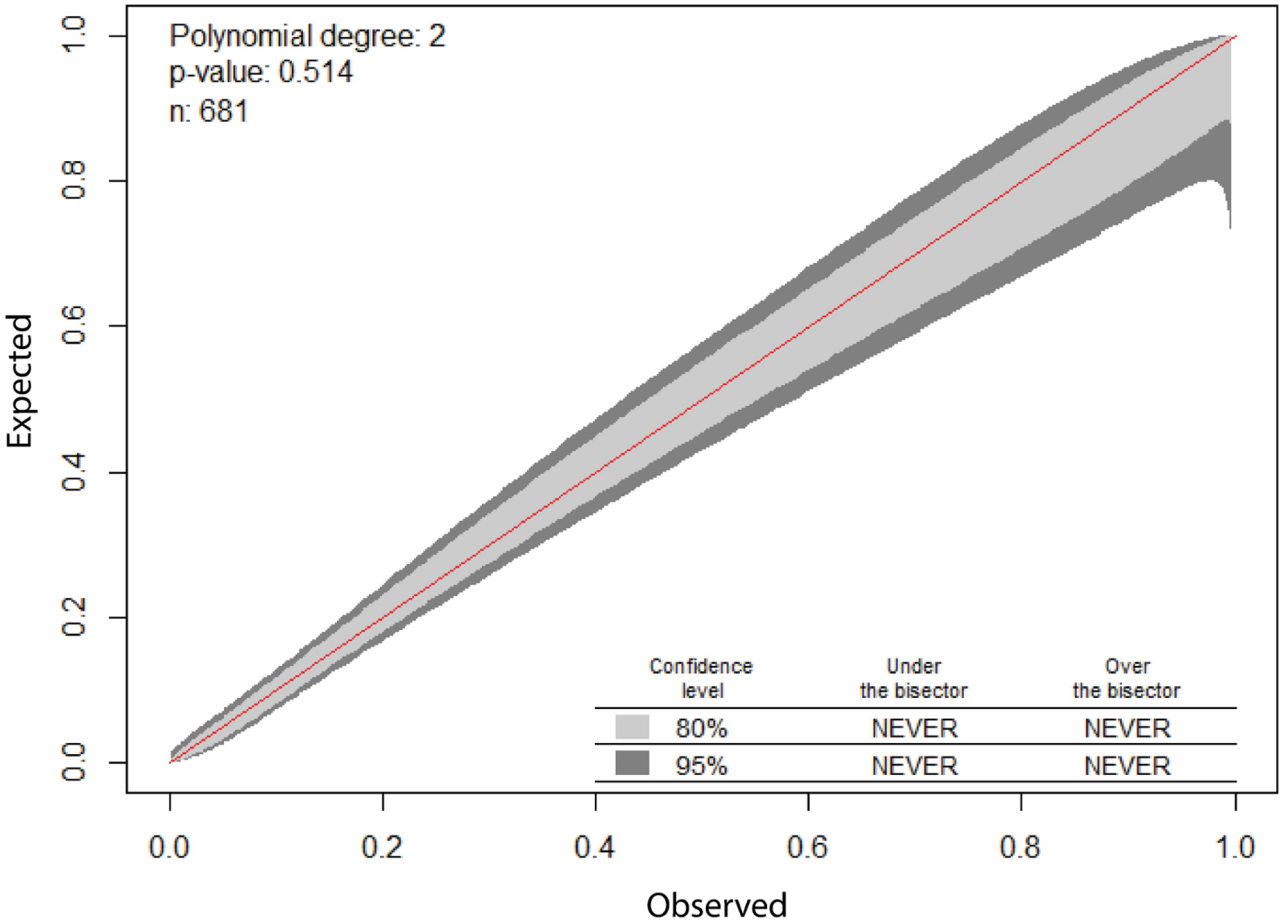
Calibration plot for admission model with initial lactate.

**Fig 4:**
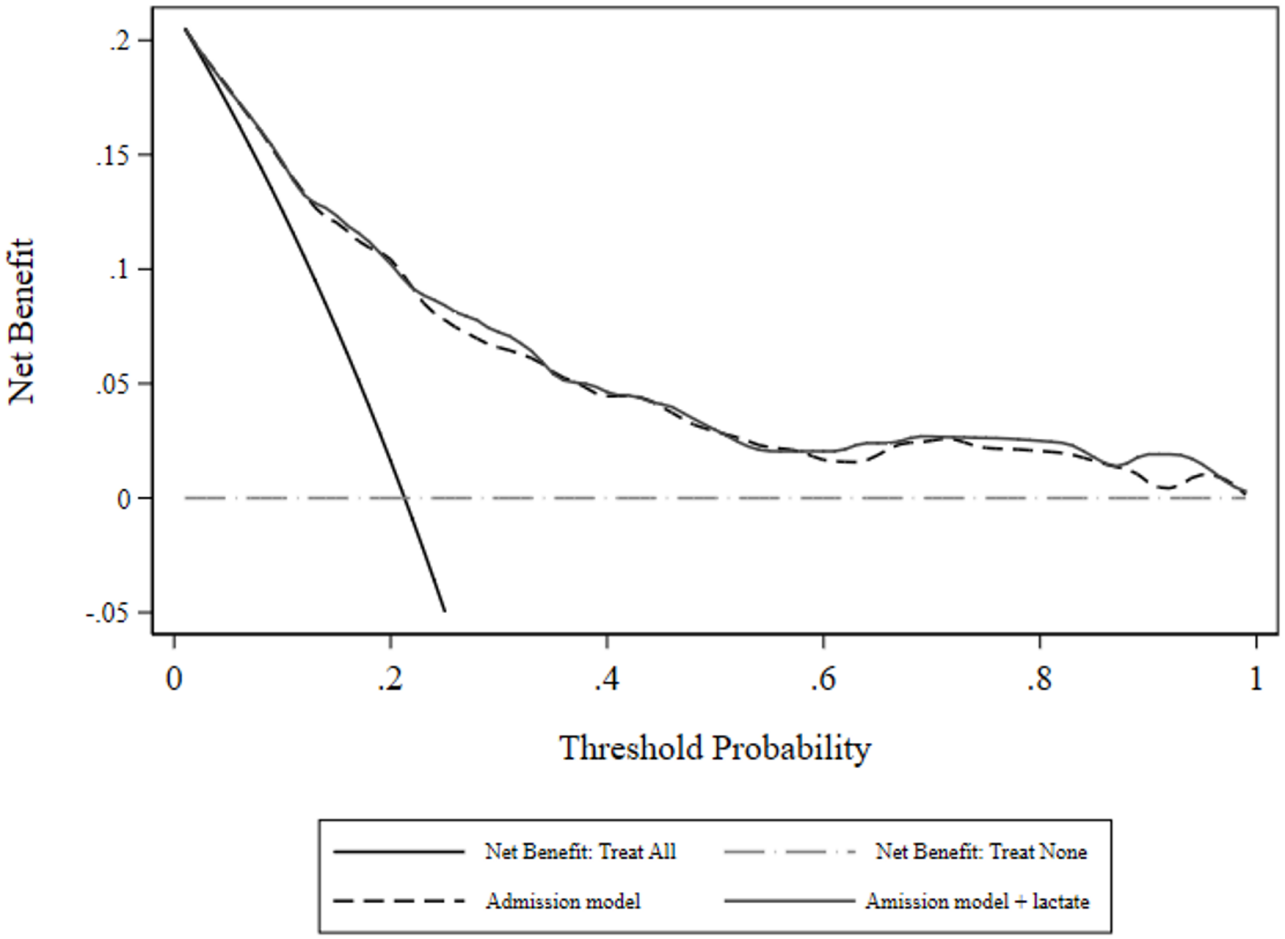
Decision curve analysis: Net benefit for admission model, with and without initial lactate.

The best fitting model (n = 681 evaluable patients) incorporated age and initial lactate (linear effects), index of comorbidity (as a 0.5, 3 fractional polynomial) and categorical variables indicating coma, cirrhosis and a heart rate ≥ 150 beats per minute. Model parameter estimates, diagnostics and risk reclassification measures are seen in Table 2. The calibration line of identity was contained within the 80 and 95% CI over the whole range (Fig 3). Measures of net benefit are shown in Fig 4 and it is obvious that despite lactate being an “independent predictor” of hospital outcome, there was little or no overall net benefit of including it in a predictive model, although both models had net benefit across all threshold probabilities.

**Table 2:** Model parameter estimates, diagnostics and risk reclassification measures: Initial lactate.

	Odds Ratio	P	Lower 95%CI	Upper 95% CI
Age (years)	1.032	0.000	1.015	1.048
CCI: FP 0.5	0.199	0.023	0.049	0.800
CCI: FP 3	40.045	0.000	13.043	122.948
Cirrhosis	3.669	0.004	1.497	8.990
Coma	80.901	0.000	14.846	440.857
MPM_O_HR	5.680	0.002	1.854	17.403
Lactate	1.127	0.005	1.038	1.224
Year 2	1.044	0.917	0.465	2.343
Year 3	0.627	0.219	0.298	1.320
Year 4	0.913	0.803	0.447	1.867
Year 5	0.400	0.013	0.194	0.824
Year 6	0.449	0.036	0.213	0.949
Year 7	0.387	0.058	0.145	1.034
**Model diagnostics**				
Hosmer-Lemeshow statistic	0.580			
AUC-ROC	0.785(0.727, 0.819)			
Condition number	11.8			
In-sample bias	0.97%			
Over-fitting	7.8%			
Out-of-sample-bias	8.7%			
	Estimate	P	Lower 95%CI	Upper 95% CI
**NRI(>0)**				
Event	-0.241		-0.375	-0.071
Non-event	0.481		0.370	0.577
Overall	0.240		0.030	0.464
**IDI**				
Event	0.007		-0.0001	0.030
Non-event	0.002		-0.0001	0.008
Overall	0.010		-0.0001	0.038
AUC-ROC difference	0.003	0.061	-0.004	0.016

CCI, Charlson comorbidity index. FP, fractional polynomial. HR, heart rate. H-L, Hosmer-Lemeshow

AUC-ROC, area under the receiver operator characteristic curve. diff, difference (model with and without lactate). NRI(>0), category free net reclassification index. IDI, integrated discrimination improvement index.

**Fig 5:**
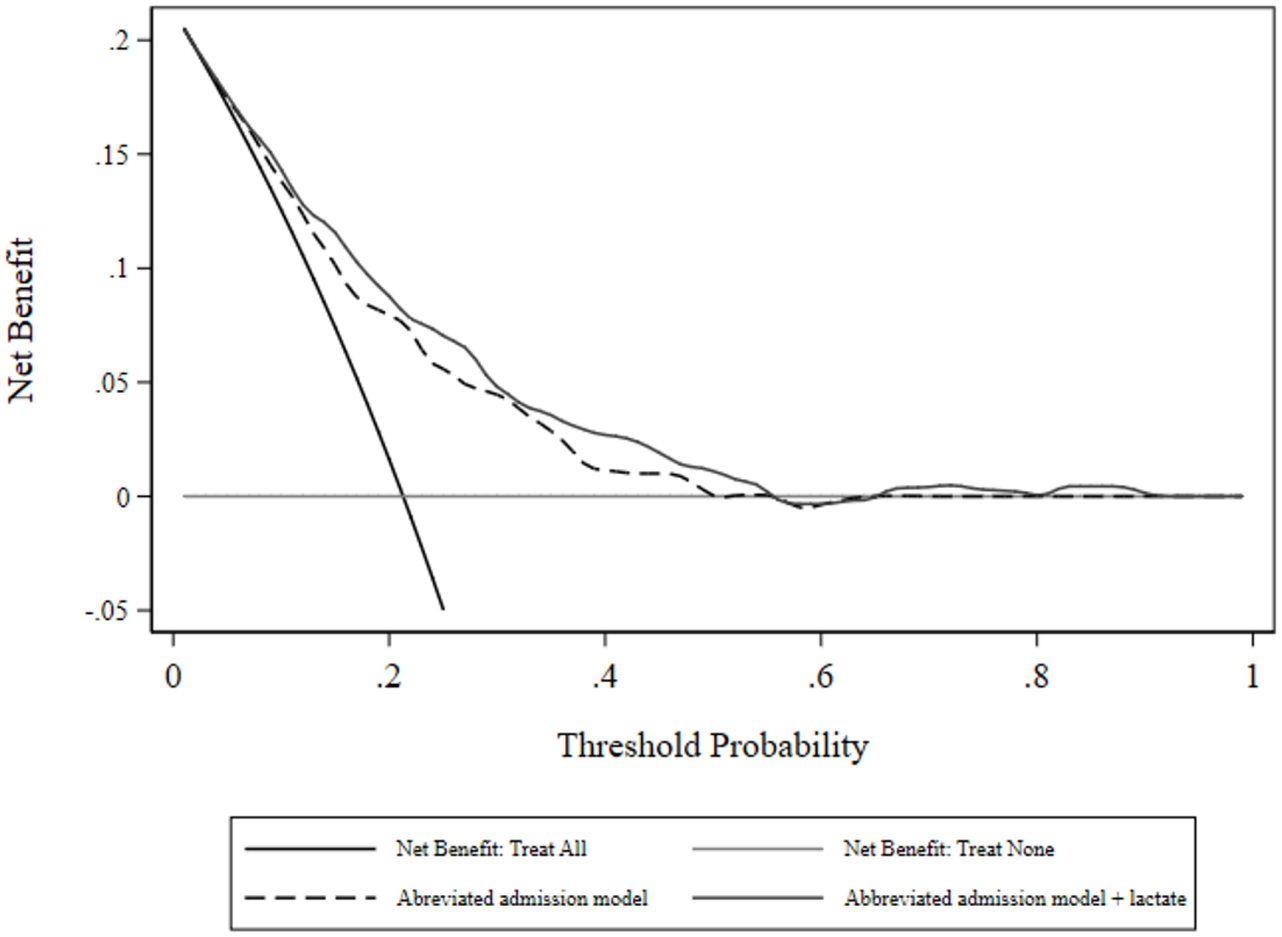
Decision curve analysis: Net benefit for abbreviated admission model, with and without initial lactate.

As a sensitivity analysis with respect to the added value of a biomarker in a “poorly” performing model [56], two categorical predictors above were dropped (coma and a heart rate ≥ 150 beats per minute) and the logistic analysis was repeated. Model parameter estimates, diagnostics and risk reclassification measures are seen in Table 3. Measures of net benefit are shown in Fig 5; there was some separation of the two curves with (small) advantage to inclusion of lactate as predictor, but no benefit of either models beyond a threshold probability of approximately 0.58.

**Table 3:** Model parameter estimates, diagnostics and risk reclassification measures.

	Odds Ratio	P	Lower 95%CI	Upper 95% CI
Age (years)	1.026	0.001	1.011	1.042
CCI: FP 0.5	0.232	0.032	0.061	0.882
CCI: FP 3	23.086	0.000	8.356	63.784
Cirrhosis	3.259	0.008	1.371	7.750
Lactate	1.201	0.000	1.120	1.289
Year 2	1.058	0.885	0.494	2.266
Year 3	0.639	0.214	0.315	1.296
Year 4	0.920	0.809	0.467	1.813
Year 5	0.433	0.016	0.220	0.854
Year 6	0.407	0.016	0.196	0.848
Year 7	0.482	0.114	0.195	1.191
**Model diagnostics**				
Hosmer-Lemeshow statistic	0.770			
AUC-ROC	0.740(0.675, 0.777)			
	Estimate	P	Lower 95%CI	Upper 95% CI
**NRI(>0)**				
Event	-0.186		-0.316	-0.026
Non-event	0.604		0.467	0.657
Overall	0.418		0.185	0.587
**IDI**				
Event	0.031		0.009	0.066
Non-event	0.008		0.002	0.018
Overall	0.040		0.0120	0.084
AUC-ROC difference	0.033	0.031	0.008	0.065

CCI, Charlson comorbidity index. FP, fractional polynomial. HR, heart rate. H-L, Hosmer-Lemeshow

AUC-ROC, area under the receiver operator characteristic curve. diff, difference (model with and without lactate). NRI(>0), category free net reclassification index. IDI, integrated discrimination improvement index.

#### Dynamic lactate indices

Both the scatter plot of fractional clearance against initial lactate and Kaiser’s R (= 0.322) favoured fractional clearance over lactate change. However, the minimum slope of the reduced major axis (= 1.112) of log lactate_initial_-log lactate_final_ suggested efficacy for the log lactate ratio which was also considered.

### Multivariate analysis: Overtime variables, fractional clearance

**Fig 6:**
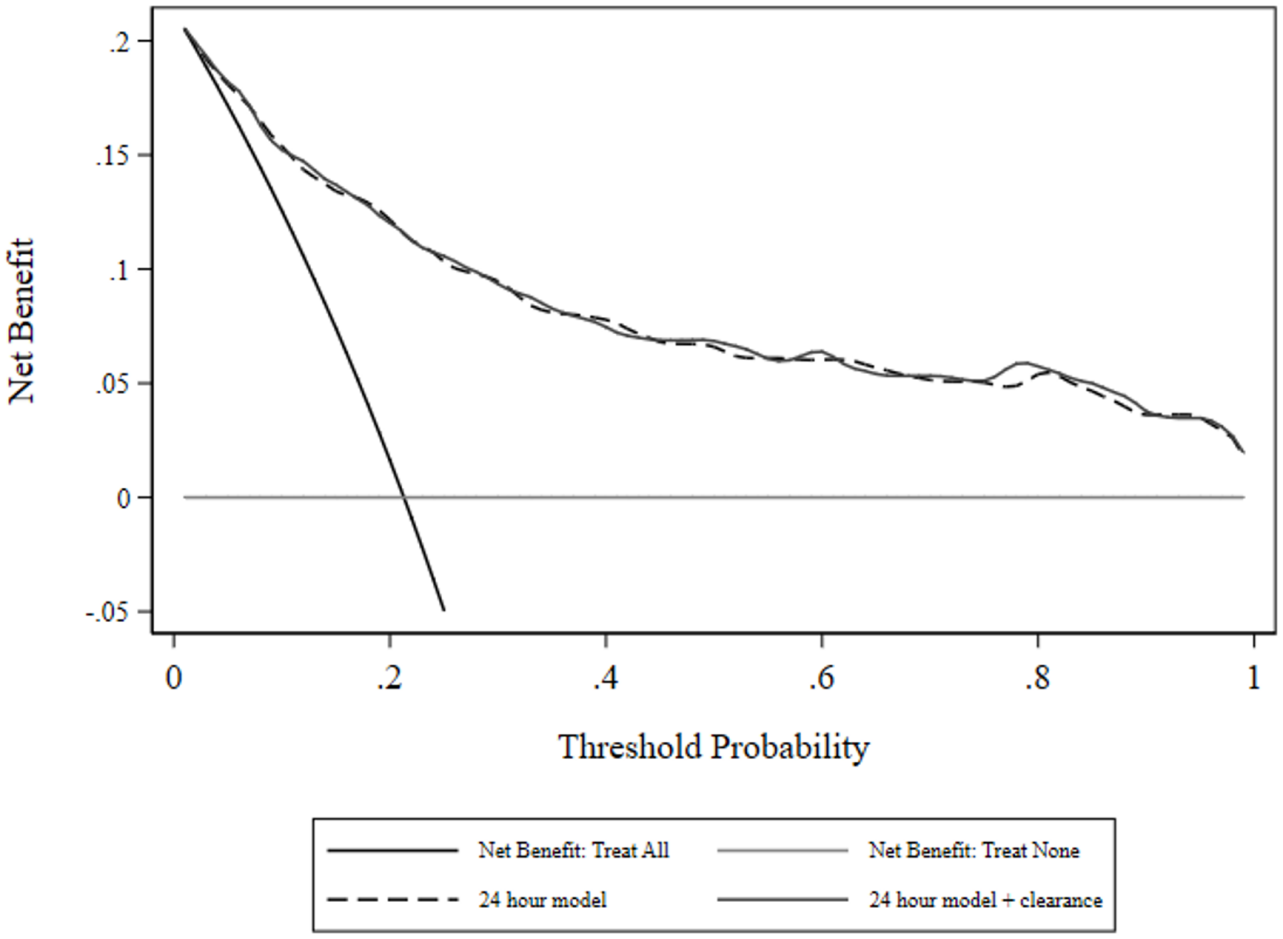
Decision curve analysis: Net benefit for 24-hour model, with and without clearance.

The best fitting model (n = 662 evaluable patients) incorporated age and clearance (linear effect), index of comorbidity (as a 0.5, 3 fractional polynomial), APACHE III score (third-degree fractional polynomial) and categorical variables indicating coma and cirrhosis (the variable denoting heart rate ≥ 150 beats per minute was non-significant at P = 0.123 and was removed from the model with no change of information criteria). Model parameter estimates, diagnostics and risk reclassification measures are seen in Table 4; lactate clearance was non-significant. The calibration line of identity was contained within the 80 and 95% CI over the whole range (S1 Fig). Model measures of net benefit are shown in Fig 6 and there was little or no overall net benefit of including clearance in a predictive model, although both models had net benefit across all threshold probabilities, of greater magnitude than the admission models.

**Table 4:** Model parameter estimates, diagnostics and risk reclassification measures: Lactate clearance.

	Odds Ratio	P	Lower 95%CI	Upper 95% CI
Age (years)	1.017	0.057	1.000	1.035
CCI: FP 0.5	0.237	0.047	0.057	0.983
CCI: FP 3	28.496	0.000	8.336	97.411
APACHE III score: FP 3	7.182	0.000	4.181	12.337
Coma	11.646	0.021	1.458	93.036
Cirrhosis	2.955	0.029	1.118	7.809
Clearance	0.771	0.078	0.578	1.030
Year 2	1.433	0.417	0.602	3.412
Year 3	0.829	0.658	0.361	1.905
Year 4	1.053	0.898	0.478	2.321
Year 5	0.472	0.067	0.211	1.055
Year 6	0.623	0.277	0.266	1.462
Year 7	0.377	0.098	0.119	1.197
**Model diagnostics**				
Hosmer-Lemeshow statistic	0.470			
AUC-ROC	0.838(0.784, 0.865)			
Condition number	11.8			
In-sample bias	1.45%			
Over-fitting	5.6%			
Out-of-sample-bias	6.9%			
	Estimate	P	Lower 95%CI	Upper 95% CI
**NRI(>0)**				
Event	-0.135		-0.310	0.217
Non-event	0.244		-0.112	0.416
Overall	0.109		-0.193	0.425
**IDI**				
Event	0.002		-0.003	0.016
Non-event	0.001		-0.001	0.004
Overall	0.002		-0.0030	0.020
AUC-ROC difference	0.001	0.78	-0.003	0.014

CCI, Charlson comorbidity index. FP, fractional polynomial. H-L, Hosmer-Lemeshow

AUC-ROC, area under the receiver operator characteristic curve. diff, difference (model with and without clearance). NRI(>0), category free net reclassification index. IDI, integrated discrimination improvement index.

A second sensitivity analysis was performed, restricting the lactate time span (admission to last) to ≥ 6 hours; the clearance estimate was OR 0.777, 95%CI: 0.583, 1.037. The decision curve analysis graph of net benefit (24-hour model versus 24 hour model plus clearance) was unchanged (S2 Fig).

### Multivariate analysis: Overtime variable, area under the lactate-time curve

The same base model as above for lactate clearance analysis was used. Area under the lactate-time curve (n = 603 evaluable patients) was non-significant at OR 0.997, 95%CI: 0.989, 1.005, P = 0.49. Model parameter estimates, diagnostics and risk reclassification measures are seen in Table 5. The calibration line of identity was contained within the 80 and 95% CI over the whole range (S3 Fig). Net benefit analysis revealed little or no advantage of including area under the lactate-time curve in a predictive model, the graph being similar to that of the clearance analysis (S4 Fig).

**Table 5:** Model parameter estimates, diagnostics and risk reclassification measures: AUC-lactate.

	Odds Ratio	P	Lower 95%CI	Upper 95% CI
Age (years)	1.010	0.278	0.992	1.029
CCI: FP 0.5	0.284	0.068	0.073	1.097
CCI: FP 3	33.894	0.000	9.423	121.918
APACHE III score: FP 3	7.337	0.000	3.991	13.488
Coma	12.620	0.031	1.258	126.555
Cirrhosis	2.308	0.107	0.834	6.382
AUC-lactate	0.997	0.494	0.989	1.005
Year 2	1.484	0.397	0.595	3.703
Year 3	0.820	0.661	0.339	1.986
Year 4	1.018	0.966	0.440	2.360
Year 5	0.481	0.087	0.207	1.114
Year 6	0.641	0.333	0.261	1.576
Year 7	0.404	0.124	0.127	1.282
**Model diagnostics**				
Hosmer-Lemeshow statistic	0.180			
AUC-ROC	0.823(0.766, 0.840)			
Condition number	6.7			
In-sample bias	1.03%			
Over-fitting	4.9%			
Out-of-sample-bias	6.03%			
	Estimate	P	Lower 95%CI	Upper 95% CI
**NRI(>0)**				
Event	0.328		-0.349	0.468
Non-event	-0.217		-0.284	0.352
Overall	0.111		-0.222	0.403
**IDI**				
Event	-0.0001		-0.004	0.008
Non-event	-0.0001		-0.001	0.002
Overall	0.002		-0.0010	0.010
AUC-ROC difference	-0.002	0.340	-0.004	0.005

CCI, Charlson comorbidity index. APIII, APACHE III. FP, fractional polynomial. AUC-lactate, area under the lactate-time curve. H-L, Hosmer-Lemeshow AUC-ROC, area under the receiver operator characteristic curve. diff, difference (model with and without area under the lactate-time curve). NRI(>0), category free net reclassification index. IDI, integrated discrimination improvement index.

Log lactate ratio, when added to the base model above was non significant (OR 1.349, 95%CI: 0.892, 2.040; P = 0.156) and the net benefit curves were again almost coincident (graph not shown).

## Discussion

In agreement with prior reports [10, 11, 13, 57], the present study has demonstrated that initial lactate concentration, lactate clearance and area under the lactate-time curve were significant univariate predictors of hospital outcome. Estimates of AUC-ROC for lactate dependent indices were consistent with those of Puskarich et al [57], where it was clear that estimates were for a univariate analysis. Thus the cautions of Nguyen et al [3] regarding the magnitude of the AUC-ROC as being “unexpectedly low” are misplaced, as the comparator paper of Nichol et al [12], examining “critically ill patients”, showed similar unadjusted AUC-ROC estimates, but larger adjusted estimates, and these estimates were also consistent with the adjusted estimates in the current paper. However, the assessment of the lactate dependent indices by the AUC-ROC belies the quite small net benefit derived from a decision curve analysis (Fig 2), given that each of the indices was well calibrated.

Analysis using a single biological measurement will be subject to random measurement error and the (regression) coefficient estimate will be biased to the null (regression dilution bias). Repeated measurement, as in the area of the lactate-time curve, would be, prima facie, the preferred measurement variable [58]. Similarly, a variable measuring time change (or “change scores”) will be subject to regression to the mean. The two change indices, - - fractional lactate clearance and log lactate ratio - -, showed a marginal relation to initial lactate, but this would not exclude confounding by regression to the mean [41, 59]. We found little evidence for the superiority of the lactate time curve in this analysis. Of some interest, in the Rivers trial ([5], page 1373 Table 2) the area under the curve of lactate between treatment arms over the first 6 hours of therapy was non-significant (P = 0.62), compared with a significant difference in lactate clearance ([Lactate^ED presentation^ − Lactate^Hour 6^]*100/ Lactate^ED presentation^), survivors versus non-survivors (38% versus 12%, P = 0.005), in a convenience cohort of patients with severe sepsis or septic shock as reported by Nguyen, Rivers and co-workers (2004) [39].

Previous multivariate analyses have used a variety of modelling approaches to ascertain the added value of lactate; ranging from a focus on an ensemble of specific lactate indices with or without other predictive variables [10, 11, 39, 60] to a formal approach to model building [13], as undertaken in this paper. We were careful to distinguish between an admission model and models derived from over-time variables. In the initial admission model with the addition of lactate, the NRI was modest at 0.24, although the AUC-ROC difference was non-significant and the differential net benefit (without and with lactate) was negligible.

Of more import, with deletion of two covariates, a poorly performing model (in terms of the scalar value of the AUC-ROC) produced a statistical (P = 0.03) difference in the AUC-ROC with addition of lactate and a substantive increase in the NRI (0.240 to 0.418), with the major re-classification occurring in the non-event category, but no discernible difference in net benefit. Neither of the over-time multivariate models, starting with a base model AUC-ROC of 0.86, produced significance in lactate indices, differences in AUC-ROC or net benefit, although the level of net benefit from threshold probabilities 0.4–1 was greater than 0.05 compared with the admission model. These observations are consistent with studies showing that the ability of a biomarker to add value to an existing model will depend upon the existing performance (value increments will be easier in poorly performing models [56, 61]) and the metric of assessment [62].

Reports on the added value of lactate in sepsis have used AUC-ROC differences almost exclusively; but the inherent problem with this strategy is the clinical interpretability of (small) difference in AUC-ROC and what level of difference is meaningful [63]. This is exemplified in the papers addressing the new 3rd International Definition of Sepsis. Despite referencing the TRIPOD statement, the paper of Seymour et al [64] used AUC-ROC estimates and differences as the sole instrument for adjudging predictivity / discrimination. The “Explanation and Elaboration” paper of the Tripod statement [21] canvassed in some detail the use of both risk reclassification (NRI) and decision curve analysis [21] in multivariate prediction models. In an interesting response to queries regarding the 3rd International Definition of Sepsis from Makam and Nguyen [65] on the use of NRI, and Gerdin and Baker [66] on calibration of qSOFA “in various settings with other models”, Seymour and Angus [67] agreed on the advantages of the NRI in outcome prediction but suggested that “calibration is not a priority for this exercise”. Apart from the Seymour paper [64], few assessments report concomitant calibration of baseline or extended models, presumably on the basis that calibration was not determinate in answering the question at hand. This, however, is not the case. The susceptibility of NRI to increments with poorly fitting risk models has been well described [68, 69]. Both simulation and case studies have demonstrated that the general effect of miscalibration was to decrease net benefit and a miscalibrated baseline model may result in a marker having inflated utility [54, 70]. We were at pains to investigate both shrinkage statistics (in-sample bias, overfitting and out-of-sample bias) and formal calibration plots; all models were well calibrated and shrinkage statistics were at quite acceptable values (all < 10%).

Decision curve analysis and the concept of net benefit have not been previously applied to the study of lactate indices as septic risk markers. As net benefit incorporates both true positives and false positives, it can be used to compare models across a range of probability thresholds and is informative as to clinical value [55]. The study by Collins and Altman of cardiovascular risk model comparisons [53] is a good example of such use of net benefit.

We were unable to demonstrate increments of net benefit for lactate as a sepsis risk biomarker, in either univariate or multivariate settings; a finding that is akin to the conclusion of the meta-analysis of Zhang and Xu [71], that in “…[in] sepsis or septic shock, LC [lactate clearance] was of limited value in predicting mortality”. The inference underlying observational studies of high initial or an over-time decrease in lactate is that these states are discriminate between those who live or die. However, as these observational values occur under changing conditions of treatment exposure, an equally valid interpretation would be that those patients who live demonstrate decreases in lactate (over time) and those who die, do not. These two statements are not necessarily consonant. The first suggests that lactate is on the direct causal pathway between treatment and outcome; the latter, that lactate merely reflects underlying pathophysiological processes, perhaps even, an innocent bystander. To wit, the use of dichloroacetate to directly reduce lactate (≥ 20%) with no improvement in survival [72].

The two randomised controlled trials which have addressed the issue of lactate-guided therapy have also not resolved the question. Jones and co-workers [73] showed non-inferiority between lactate clearance and central venous oxygen saturation (Δ = -10% in-hospital mortality) as early sepsis resuscitation goals and found no differences in administered treatments in the first 72 hours. Jansen and co-workers [74], in critically ill patients, targeting a decrease in lactate by ≥ 20% per 2 hours for the initial 8 hours of ICU stay, found no difference in hospital mortality on the unadjusted analysis (P = 0.067), with the lactate group receiving more fluids and vasodilators. The adjusted in hospital mortality was a substantial 22% less (RR 0.78 to 0.61) and significant at P = 0.006; but it is instructive to note that neither the unadjusted or adjusted 28-day mortality was significant (P = 0.30 and 0.134, respectively). Since the classic 1988 study of Jencks et al [75] it has been known that there is a bias in hospital mortality, due to discharge practice, and this has been more recently reaffirmed [76, 77]. Thus, a more robust endpoint for both trials would have been a fixed 28 or 30 day or longer (out of hospital) mortality endpoint.

The current study proceeded from a modest sample size and did not formally address the utility of lactate with admission values ≥ 4 mmol/l, as in the Rivers trial [5], on the basis that only 17% of the patients had such elevation of lactate, although such a cut -point would appear to be more arbitrary than optimal [78]. We considered lactate as both arterial and venous. The percentage of venous specimens was 18–22%, depending upon the data-set; 8% of lactate specimens had no label. A 3-level nominal categorical variable (“blood type”) was entered into each of the regression models (initial lactate, lactate clearance and AUC-lactate). The p-values of the parameters of this variable were always ≥ 0.1. Similarly, the p-values for interaction between “years” and “blood type” was ≥ 0.13. Lactate values below 4 mmol/l were associated with increased mortality in this study (Fig 1) and others [79–81]. Our ability to test a lactate clearance over the first 6 hours was also limited by this end-point being unavailable for all patients. We would agree with the sentiments of Moons and co-workers that “Researchers and physicians should recognize, however, that a single summary measure cannot give full insight in all relevant aspects of the added, clinical value of a new test or biomarker” [22]. The inability to rank the added-value analyses is a potential weakness of this study, albeit the TRIPOD statement [21] offered no direct advice on this, which would require comparative power analyses of the various estimators. That the base models were derived and tested on the same patient cohort potentially inflated the performance characteristics [82] and may have underestimated lactate added value. Missing data occurred at each stage of the three substantive multivariate analyses; with values of 5% (admission model), 7.7% (overtime model with clearance) and 16% (overtime model with area under the lactate-time curve). Only the missing value percentage for the area under the lactate-time curve would appear problematic. This being said, complete record logistic regression may be more robust to missing values than previously assumed [83]. That our results may reflect the case-mix of a single tertiary Australian ICU is not contested, but we must be aware that all “clinical studies that use observational databases can be sensitive to the choice of database” [84].

We conclude that the ability to demonstrate lactate as a sepsis risk biomarker depends upon the performance of the underlying base model and any such demonstration must embrace other assessments of added value such as risk reclassification and net benefit. Current lactate markers, in particular, initial lactate and lactate clearance, may be subject to regression dilution and regression to the mean.

## Supporting information

S1 Fig Calibration plot for 24-hour model with clearance. (DOCX)

S2 Fig Decision curve analysis: Net benefit for 24-hour model with lactate time span (initial to final) ≥ 6 hours. (DOCX)

S3 Fig Calibration plot for 24-hour model with area under lactate time curve. (DOCX)

S4 Fig Decision curve analysis: Net benefit for 24-hour model, with and without area under lactate-time curve. (DOCX)
