# Supplementary material for: Reconsidering lactate as a sepsis risk biomarker
Source: PLoS One. 2017 Oct 3;12(10):e0185320. doi: 10.1371/journal.pone.0185320 (PMC5626033; doi:10.1371/journal.pone.0185320)
Supplement: S4 Fig — (DOCX) [file pone.0185320.s004.docx]

S4 Fig. Decision curve analysis: net benefit for 24 hour model, with and without area under lactate-time curve
